# Exposure source prevalence is associated with gender in hepatitis C virus patients from Rio de Janeiro, Brazil

**DOI:** 10.1590/0074-02760160553

**Published:** 2017-09

**Authors:** Daniele Blasquez Olmedo, Patrícia Marraccini Precioso, António Lugdero-Correia, Guida da Silva, Angela Maria Guimarães dos Santos, Luís Cristóvão Pôrto

**Affiliations:** 1 Universidade do Estado do Rio de Janeiro, Laboratório de Histocompatibilidade e Criopreservação, Rio de Janeiro, RJ, Brasil; 2 Secretaria Municipal de Saúde do Rio de Janeiro, Rio de Janeiro, RJ, Brasil

**Keywords:** risk behaviour, transmission, hepatitis C, epidemiological surveillance

## Abstract

**BACKGROUND:**

Hepatitis C virus (HCV) infection is a worldwide public health problem. A characterisation of the differences in exposure sources among genders will enable improvements in surveillance actions.

**METHODS:**

Exposure data were obtained for 1180 confirmed HCV cases Brazil’s mandatory reporting to epidemiological surveillance, which was directed by a reference laboratory in Rio de Janeiro, Brazil. The Chi-square test (χ2) was used to assess the associations between exposure sources and gender. The prevalence ratio (PR) was calculated for exposures that showed an association.

**RESULTS:**

The results showed 57.7% cases were female, and associations with snorting drugs, sexual activity, surgery, aesthetic procedures, blood transfusions, and educational level were observed (p < 0.001). Men showed 2.53 (1.33-3.57), 4.83 (3.54-6.59), and 2.18 (1.33-3.57) times more exposure to sniffing drugs, risky sex and higher levels of education, respectively, than women. Women demonstrated 4.46 (3.21-6.21), 1.94 (1.43-2.63), and 3.10 (2.09-4.61) times more exposure to surgery, aesthetic procedures, and blood transfusions, respectively, than men.

**CONCLUSION:**

Our results showed differences in risk behaviours associated with gender among HCV carriers. These data are likely to significantly influence clinical practice regarding the adoption of specific approaches for counselling and control policies to prevent the emergence of new cases and break the chain of transmission of the virus.

Important variations have been observed in the epidemiology of the hepatitis C virus (HCV) infection, including the prevalence of distinct risk factors around the world (Lavanchy 2009, Gower et al. 2014, Cullen et al. 2015, El-Ghitany et al. 2015, Muñoz et al. 2015). Recently, Gower et al. (2014) found a global prevalence of anti-HCV seropositivity of 1.6% (1.3-2.1%) among all age groups, which corresponded to 115 (92-149) million cases of past viraemic infections. The viraemic prevalence (RNA positivity) was forecasted to be 1.1% (0.9-1.3%), corresponding to 80 (64-103) million viraemic infections. Brazil ranked 8th among the countries that were studied (Gower et al. 2014).

Brazil is a continental country with a high geographical, social and cultural diversity. In a nationwide survey conducted in urban populations, the overall prevalence of anti-HCV seropositivity was 1.38% (1.12-1.64%), and predictors of HCV infections were shown to include older age, use of injected [odds ratio (OR) = 6.65%] and inhaled (OR = 2.59%) drugs, hospitalisation (OR = 1.90%), socially disadvantaged groups that lacked sewage disposal (OR = 2.53), and the use of glass syringes (OR = 1.52%). However, the known risk factors explain less than 50% of the infected cases, limiting prevention strategies (Pereira et al. 2013). The Brazilian Information System for Notifiable Diseases (SINAN) provides data to the Health Ministry (MS 2012), which issues epidemiological reports that guide public policies. HCV infection is a notifiable disease in the SINAN (Naveira et al. 2014, MS 2015). In 2014, its seroprevalence was estimated to be 1.4 million cases in Brazil. The main route of transmission is parenteral (MS 2015). The case distributions by age group and gender observed in the Southeast (SE) suggested the existence of distinct parenteral exposure patterns between the genders.

The development of direct-acting antivirals (DAAs) has introduced a new reality to HCV infection. With the safety and efficacy achieved by new therapies (i.e., DAAs), the main solutions to HCV eradication involve prevention, control, and diagnosis (Hagan & Schinazi 2013, Wedemeyer et al. 2014) which, when applied together, can substantially reduce (> 50%) the prevalence of HCV among high-risk individuals (Hagan et al. 2011, Martin et al. 2013). A good understanding of the routes of transmission is required to develop strategies to eliminate the HCV infection (Martin et al. 2013, Wedemeyer et al. 2014).

The World Health Organization (WHO 2000) recognises that there is a clear need for surveillance of transmissible diseases (TD), especially those with epidemic potential, such as HCV infection (Sharp et al. 2014, Seale et al. 2015). However, early detection should not be the only action of surveillance when the aim is its eradication. Recognising the main risk factors is essential, but recognising any exposure source pattern in those who carry the aetiologic agent can provide information regarding vulnerabilities to the appearance of secondary cases and can indicate possible fragilities in control systems. While some studies have sought to evaluate the exposure sources in men having sex with men (MHM) and people who inject drugs (PWID) (Martin et al. 2013, Fernández-Dávila et al. 2015, Marcellin et al. 2015), there is no direct evaluation of the exposure patterns that affect men and women that are important to individualised counselling and the disruption of the chain of transmission.

To assess which risk behaviours between genders may lead to continuous transmission of the HCV and develop recommendations for their management, we carried out a study of the association between exposure patterns in confirmed cases using data from mandatory notification.

## MATERIALS AND METHODS

*Subject enrolment and data collection* - The present study was a descriptive, retrospective study of a series of cases that were followed for molecular HCV detection through viral load quantification and/or genotyping tests, which were performed at the Histocompatibility and Cryopreservation Laboratory of Rio de Janeiro State University (UERJ-HLA). The cases were considered as confirmed when the patient presented detectable HCV ribonucleic acid (RNA) or undetectable HCV-RNA through treatment. Non-confirmed cases were excluded from the study.

The data in the analysis were extracted from a database of all HCV cases submitted for laboratory surveillance from January 2013 to August 2015 at UERJ-HLA. The laboratory surveillance was conducted by a routine implemented as a new checkpoint for case notification control by the city of Rio de Janeiro Health Secretary. Briefly, patients with an HCV molecular test prescription that consented to participate were subjected to extensive epidemiological interviews that occurred before their blood collections. The data were registered on the official surveillance form of the Health Ministry used for case notification. The data entries were compiled in a customised Microsoft Access® database after data completeness revisions by the investigators. The form included socio-demographic data (age, gender, educational status, and self-declared skin colour), exposure sources throughout life or in the last six months, such as blood transfusion, transplantation, surgery and dental procedures (with bleeding), injectable medications administered with a glass syringe, inhalable and intravenous drugs, haemodialysis, acupuncture, tattooing, piercing, coinfection with human immunodeficiency virus (HIV), sexual activity (three or more sexual partners), other activities (related to nail care and barbers or other sources), accidents with biological materials, HCV viral load, and genotype. Information about hepatitis A virus (HAV) and hepatitis B virus (HBV) vaccinations was also collected through self-reports. Treatment status data were included in the forms.

*Statistical analysis* - Descriptive statistics were generated for the socio-demographic characteristics and exposure sources. Continuous variables were reported as the mean ± standard deviation (SD) or median [interquartile range (IQR), minimum-maximum], and the Chi-square test (χ^2^) for independence was used to assess the association of categorical variables and exposure source status. The prevalence ratio (PR) of each exposure source that demonstrated an association with gender was calculated with 95% confidence intervals (95% CI) and stratified by two age groups: less than or equal to 45 years of age (≤ 45) and over 45 years of age (> 45). Multivariate analysis was performed using a binary logistic regression model when a variable in the univariate model had a p-value < 0.20. The results were considered statistically significant when p ≤ 0.05. The statistical analyses were performed using the Statistical Package for the Social Sciences (SPSS for Windows, release 17.0; SPSS Inc., Chicago, IL, USA).

*Ethical issues* - The ethics committee of the Hospital Universitário Pedro Ernesto (HUPE) 139 approved the study protocol (nº 1.274.583). The procedures followed were in accordance with the ethical standards of the Helsinki Declaration (2008).

## RESULTS

*Demographic characteristics of the interviewed HCV patients* - A total of 4,897 patients were tested for HCV-RNA detection from January 2013 to August 2015 at the UERJ-HLA. Patients were interviewed at the first confirmatory test for HCV-RNA (78.9%), but many patients were interviewed during the monitoring of treatment or post-treatment evaluation. A total of 1,541 patients with suspected HCV agreed to answer the questionnaire and received an epidemiological interview, and 1,180 were confirmed cases. The characteristics of the confirmed HCV cases are shown in Table I. In all, 57.7% of the cases were female. The median age was 58 years (IQR 0-87), and there was no statistically significant difference between the mean ages of the males and females. Individuals who were 46-60 years of age comprised 45.1% of the cases (532/1180). There was no difference in the gender proportions within the age groups (p = 0.53, data not shown).

**TABLE I:** Characteristics of the hepatitis C virus (HCV) cases from January 2013 to August 2015

Characteristics	N	(%) of total	Lower	Upper	p value
Sex					
Female	681	57.7	54.9	60.5	
Male	499	42.3	39.5	45.1	< 0.001
Ethnicity mixed					
	508	43.0	40.2	45.9	
White	355	30.0	27.5	32.7	
Black	298	26.0	22.8	27.8	
Native Brazilian/Oriental	11	0.9	0.5	1.6	
Missing	8	0.1	0.3	1.3	< 0.001
Educational level					
Basic level (< 10 yr)	627	53.2	50.3	56.0	
Medium level (10 to 12 yr)	387	32.8	30.2	35.5	
Higher level (> 12 yr)	137	11.6	9.9	13.6	
Missing	29	2.4	1.7	3.5	< 0.001
Age group*					
> 75	32	3.0	1.9	3.80	
61 - 75	402	34.0	31.4	36.8	
46 - 60	532	45.0	42.3	47.9	
31 - 45	154	13.0	11.2	15.1	
16 - 30	35	2.9	2.1	4.1	
< 15	25	2.1	1.4	3.1	< 0.05
	Mean	SD			
HCV viral-load (log IU/mL)	5.7	1.0			
Genotypes (G) n = 718					
G1	619	86.2	83.5	88.5	
G2	15	2.2	1.3	3.4	
G3	78	10.8	8.8	13.3	
G4	6	0.8	0.4	1.8	< 0.001

*: age group by interquartile range (IQR); median age 58 (0-87) and mean age standard deviation (SD): 55.05 (13.84). yr: years; N: number of cases; IU/mL: international units by milliliters statistically significant when p ≤ 0.05.

Individuals of mixed and white self-declared colour/race were the major groups, representing 73.1% of the cases. Only 11.6% of the HCV cases had at least 12 years of education. HCV genotype 1 was present in 88.2% of the cases, and in those who were treatment-naive (827), the mean viral load was 5.7 (± 1.0) Log IU/mL.

*The associations of exposure sources among the HCV cases in the univariate analysis* - Table II shows the history of exposure sources among the cases. The most prevalent exposure sources among the confirmed cases were dental and surgical treatment (93.5% and 82.4%). Exposure to sniffing drugs, illicit intravenous drugs, sexual activity, surgical treatment, blood transfusion, other exposures and HIV coinfection exhibited gender differences (p < 0.001). Exposures to inhaled drugs, injectable drugs, and sexual activity were 3.91, 4.52, and 2.15 times more prevalent, respectively, in males compared to females. In contrast, surgical procedures, other exposures, and blood transfusions were more prevalent in women by 1.28-, 1.39- and 1.62-fold, respectively. Exposure to HIV coinfection was excluded from the analysis of prevalence ratios because the amount of missing information was high (Table III).

**TABLE II:** Exposure sources of the histocompatibility and cryopreservation laboratory of Rio de Janeiro State University (UERJ-HLA) hepatitis C virus (HCV) cases interviewed from January 2013 to August 2015

Exposure sources**	Male		Female		Total		p
		
	N	(%)	N	(%)	N	(%)	
Injectable medication							
Yes	348	69.7	456	67.0	804	68.1	0.657
No	122	24.5	170	25.0	292	24.7	
Missing	29	5.8	55	8.1	84	7.1	
Sniffing drugs							
Yes	163	32.7	57	8.4	220	18.6	< 0.001
No	333	66.7	622	91.3	955	80.9	
Missing	3	0.6	2	0.3	5	0.4	
Illicit intravenous drugs							
Yes	86	17.2	26	3.8	112	9.4	< 0.001
No	410	82.2	653	95.9	1063	90.0	
Missing	3	0.6	2	0.3	5	0.4	
Sexual							
Yes	340	68.1	217	31.9	557	47.2	< 0.001
No	152	30.5	459	67.4	611	51.7	
Missing	7	1.4	5	0.7	12	1.0	
Tattoo/piercing							
Yes	90	18.0	111	16.3	201	17.0	0.419
No	406	81.4	568	83.4	974	82.5	
Missing	3	0.6	2	0.3	5	0.5	
Acupunture							
Yes	69	13.8	103	15.1	172	14.6	0.571
No	423	84.8	574	84.3	997	84.5	
Missing	7	1.4	4	0.6	11	0.9	
Surgical treatment							
Yes	353	70.7	620	91.0	973	82.4	< 0.001
No	145	29.1	60	8.8	205	17.4	
Missing	1	0.2	1	0.1	2	0.1	
Dental procedures							
Yes	464	93.0	640	94.0	1104	93.5	0.388
No	34	6.8	38	5.6	72	6.1	
Missing	1	0.2	3	0.4	4	0.4	
Haemodialysis							
Yes	19	3.8	20	2.9	39	3.31	0.407
No	479	96.0	660	96.9	1139	96.5	
Missing	1	0.2	1	0.1	2	0.2	
Other							
Yes	271	54.3	524	76.9	795	67.3	< 0.001
No	213	42.7	145	21.3	358	30.3	
Missing	15	3.0	12	1.8	27	2.2	
Blood trasfusion							
Yes	173	34.7	386	56.7	559	47.3	< 0.001
No	299	59.9	262	38.5	561	47.5	
Missing	27	5.4	33	4.8	60	5.0	
Transplantation							0.969
Yes	6	1.2	8	1.2	14	1.1	
No	492	98.6	670	98.4	1162	98.4	
Missing	1	0.2	3	0.4	4	0.34	
ABM*							
Yes	56	11.2	59	8.7	115	9.7	0.158
No	430	86.2	598	87.8	1028	87.1	
Missing	13	2.6	24	3.5	37	3.1	
HIV							
Yes	55	11.0	28	4.1	83	7.0	< 0.001
No	320	64.1	470	69.0	790	66.9	
Missing	124	24.8	183	26.9	307	26.0	

*: accident with biologic material; **: exposure sources missing data: 3.42%(565/15955); N: number of cases; Chi-square test (χ2) was used to assess the association between exposure sources and gender. Statistically significant when p ≤ 0.05.

**TABLE III:** Prevalence ratio (PR) of exposure sources between men and women infected with hepatitis C virus (HCV) by age group

Exposure sources	Gender		N	PR (95% CI)	p value

	Male (%)	Female (%)			
Sniffing drugs	74.1	25.9	220	3.91 (2.96-5.17)	< 0.001
< = 45	60.0	40.0	40	1.89 (1.07-3.36)	0.025
> 45	77.2	22.8	180	4.72 (3.41-6.52)	< 0.001
Illicit intravenous drugs	76.8	23.2	112	4.52 (2.96-6.91)	< 0.001
< = 45	63.6	36.4	11	2.21 (0.66-7.34)	0.181
> 45	78.2	21.8	101	5.98 (3.65-9.78)	< 0.001
Sexual	61.0	39.0	557	2.15 (1.90-2.43)	< 0.001
< = 45	52.5	47.5	118	1.40 (1.10-1.78)	0.005
> 45	63.3	36.7	439	2.41 (2.08-2.79)	< 0.001
Surgical	36.3	63.7	973	1.28 (1.21-1.36)	< 0.001
< = 45	37.2	62.8	148	1.33 (1.09-1.62)	0.002
> 45	36.1	63.9	825	1.27 (1.19-1.35)	< 0.001
Other	34.1	65.9	795	1.39 (1.28-1.52)	< 0.001
< = 45	34.9	64.1	149	1.46 (1.20-1.78)	< 0.001
> 45	33.9	66.1	646	1.38 (1.25-1.52)	< 0.001
Blood transfusion	30.9	69.1	559	1.62 (1.42-1.85)	< 0.001
< = 45	49.4	50.6	85	0.81 (0.58-1.11)	0.196
> 45	27.6	72.4	474	1.87 (1.60-2.18)	< 0.001

N: number of cases. PR was calculated with 95% conﬁdence intervals (95% CI). Statistically significant when p ≤ 0.05.

The exposure prevalence of inhaled and injectable drugs, blood transfusions, and sexual activity were also associated with gender in the > 45-years-old age group. Men in the > 45-years-old age group reported 4.72, 5.98, and 2.41 times more exposure to sniffing drugs, illegal intravenous drugs, and sexual activity, respectively, than women. Women in the same age group reported exposures to surgical procedures, other sources, and blood transfusions that were 1.27, 1.38, and 1.87 times higher, respectively, compared to men. The association of gender with exposure to surgical treatment and “other” sources was observed in both age groups (p < 0.001), as shown in Table III.

The education level was independently associated with gender, demonstrating a linear association (p < 0.05). The basic level was reported to be 1.33 times more for women than men, and the higher education level was 2.45 times higher in men than women (Table IV).

**TABLE IV:** Difference of educational level among the cases according gender

Educational level	Gender		N	PR (95% CI)	p value

	Male (%)	Female (%)			
Basic level	44.5	59.5	627	1.33 (1.19-1.50)	< 0.001
Medium level	33.9	32.0	387	1.05 (0.89-1.24)	0.502
Higher level	17.6	7.2	137	2.45 (1.76-3.40)	< 0.001

PR: prevalence ratio; N: number of cases. PR was calculated with 95% conﬁdence intervals (95% CI). Statistically significant when p ≤ 0.05.

*The associations of exposure sources among the HCV cases by multivariate analysis* - In the multivariate analysis, the prevalence of injectable drug use and basic education level lost their association to gender, suggesting that other factors may have acted as confounders in the univariate analysis. Exposures to inhaled drugs and sexual activity remained with a higher prevalence in men and were 2.53- and 4.83-fold higher, respectively, in men than women (Table V). Exposures to surgical treatment, other sources and blood transfusion were 4.46-, 1.94-, and 3.10-fold higher, respectively, in women than men.

**TABLE V:** Multivariate analysis of the Histocompatibility and Cryopreservation Laboratory of Rio de Janeiro State University (UERJ-HLA) hepatitis C virus (HCV)-infected cases reported to Brazilian Information System for Notifiable Diseases (SINAN) from January/2013 to August/2015

Variables	Prevalence ratio (PR)			

	Male	Female	95% CI	p
Exposure sources				
Sniffed drugs	2.53	1	1.57-4.10	< 0.001
Illicit intravenous drugs	1.52	1	4.83-3.54	0.208
Sexual	4.83	1	3.54-6.59	< 0.001
Surgical	1	4.46	3.21-6.21	< 0.001
Other	1	1.94	1.43-2.63	< 0.001
Blood transfusion	1	3.10	2.09-4.61	< 0.001
Educational level				
Basic level	1	1.16	0.85-1.60	0.340
Higer level	2.18	1	1.33-3.57	0.002

The independent association among variables, with respect to gender, was evaluated by logistic regression in order to confirm the profile of differential exposure found among the confirmed HCV cases. Multivariate analysis was performed using a binary logistic regression model when a variable in the univariate model had a p-value < 0.20. Statistically significant when p ≤ 0.05.

## DISCUSSION

Parenteral exposure is multifaceted and depends mainly on individual risk behaviours. Factors that remain unchanged across time, such as gender, provide evidence for the validity of the statistical associations. The official government data show that the total number of infected men reported to SINAN was greater than the number of women. However, their HCV detection rates were very similar, and the gender ratio decreased from 2008 to 2012, when it was 1.2 to 0.9 (each case in men was compared to 1 case of disease in women) (SES/RJ 2014). The 1:3 ratio found in this study may indicate a change in the sex ratio. Nevertheless, the gender proportion observed herein was similar to that observed in the SE region in a national survey, where women were predominant (57.7%) (Pereira et al. 2013).

In our study, the method used for the data collection permitted a low loss of exposure data (3.42%). The data integrity enabled the identification of the main exposure sources reported by a case series in the following order: dental and surgical procedures, injectable medications, other exposures, sexual risk behaviour, and blood transfusions. However, the frequency of exposure to some risk factors for HCV was more heavily reported by one gender than the other, suggesting a difference between exposure sources. Recognition of these sources can be essential to improving the actions of targeted epidemiological surveillance because knowledge of risk factors is not enough to ensure attitude.

In Brazil, extreme poverty, advanced age and injectable drugs are risk factors for HCV, but not education. However, poor education is a risk factor in the Egyptian population (Pereira et al. 2013, El-Ghitany et al. 2015). We found an association between education and HCV infection in relation to gender. Cases that had a higher level of education (> 12 years) were less common (11.6%), but there was a higher prevalence in males when analysed with other variables, where the prevalence in males was twice that observed in women. The high prevalence of cases among persons with basic and higher educational levels suggests that a clear and accessible approach should be considered in prevention and control policies.

Worldwide, people who inject drugs are known to have a high risk for HCV infection, as do blood transfusion recipients. On the other hand, the extent of this risk exposure may be under-reported due to stigmatisation and general confounders (Marinho & Barreira 2013). There was no higher exposure to the parenteral transmission sources that are most commonly associated with risk (Marcellin et al. 2015, Muñoz et al. 2015), such as drug use (inhalable = 18.6%; injectable = 9.4%), and the prevalence for exposure to blood transfusions (47.3%) was similar to that for sexual risk behaviour (47.2%), which has already been noted (Pereira et al. 2013).

The published data on sexual and intra-family transmission are inconsistent (Cavalheiro 2007). Sexual behaviour with multiple sexual partners and the increased risk levels in specific groups, such as HIV and HBV co-infected persons, sex workers, homosexuals, illicit drug users, and populations with clinically evident sexually transmitted infections (STIs) are common risk factors for HCV infection. The rate of HCV infection related to sexual transmission may range from zero to 27%. However, a review by Cavalheiro (2007) concluded that the chances of transmission were low and almost nil. Intra-familial transmission is strongly implicated and thought to be a confounder due to sharing of personal hygiene items. In the United States, the Centers for Disease Control and Prevention (CDC 2012) estimated that between 20-25% of the transmission indices were associated with sexual contact, although the worldwide figures fluctuate in different populations. In our investigation, the measure of sexual exposure was limited to the occurrence of sexual risk behaviour involving unsafe sexual practices with three or more partners. There was no evaluation regarding types of sexual intercourse (Fernández-Dávila et al. 2015). Further studies should be conducted in this area.

*Limitations* - This study has some limitations. First, compulsory notification of viral hepatitis has limited reliability even if collected through an interview. The loss of data can be minimised, but the stigma associated with HCV infection may limit the reliability of self-reported data (Marinho & Barreira 2013). Second, a case series analysis cannot determine a temporal sequence of cause and effect. Analyses in case series are easy to perform but serve to formulate hypotheses about groups of interest. Here, it was notable that men and women infected with HCV differed in their epidemiological history regarding exposures to their risk factors for HCV, but the correlations found do not imply causation. Third, a limited group analysis cannot represent the total population. Available information regarding the reference population indicates that there are 3,423 cases in the city and 6,162 in the state of RJ (SES/RJ 2014). This study was conducted in one city in Brazil, so the epidemiological conclusion may be very limited to exposure sources from a specific population even though it is based on known global risk factors for HCV infection. Finally, the high proportion of people who were 45 years of age or older represents the exposure profiles of baby boomers, and the exposure sources of these men and women cannot represent exposure sources in younger men and women.

Adults born between 1945 and 1965 are more likely to be diagnosed with the HCV infection if they received a blood transfusion before the introduction of HCV screening in 1992 (CDC 2012). Based on these data, the CDC and the Ministry of Health of Brazil established a control policy for blood and recommend testing for this age group (Naveira et al. 2014). As most of the patients in our analysis were older (> 45 years), our analysis may have been influenced by the high prevalence of cases in this age group.

Finally, our experience suggests that the harm reduction policy for men should focus on condom distribution, paraphernalia, and pipes for drug users, but for women, direct surveillance information regarding the safety of surgical procedures, such as aesthetic procedures or the use of sharp blades, and blood transfusions should be a very useful and effective approach towards infection control. There is no doubt that for both genders, the empowerment of information regarding the main routes of transmission and the power of choice will create a range of opportunities for preventing and reducing infection in the population.

*Conclusions* - Our results showed differences in risk behaviour that was associated with gender among HCV carriers. The behaviours that expose individuals to known risk factors for HCV infection, such as drug use, blood transfusions, unprotected sex with multiple partners and surgical treatments, appear to be differentially associated with gender. The implementation of routine epidemiological interviews and counselling following notification may be helpful to support the surveillance of HCV and provide information for population studies. Knowledge of these risk behaviours should contribute to the eradication of the disease through prevention and control to break the chain of virus transmission.
